# Prevalence of Olfactory Dysfunction with the Omicron Variant of SARS-CoV-2: A Systematic Review and Meta-Analysis

**DOI:** 10.3390/cells12030430

**Published:** 2023-01-28

**Authors:** Christopher S. von Bartheld, Lingchen Wang

**Affiliations:** 1 Department of Physiology and Cell Biology, Reno School of Medicine, University of Nevada, Reno, NV 89557-0352, USA; 2 School of Public Health, University of Nevada, Reno, NV 89557-0275, USA

**Keywords:** omicron, SARS-CoV-2, COVID-19, anosmia, loss of smell, prevalence, ethnicity, host factor, UGT2A1, UDP glycosyltransferase

## Abstract

The omicron variant is thought to cause less olfactory dysfunction than previous variants of SARS-CoV-2, but the reported prevalence differs greatly between populations and studies. Our systematic review and meta-analysis provide information regarding regional differences in prevalence as well as an estimate of the global prevalence of olfactory dysfunction based on 62 studies reporting information on 626,035 patients infected with the omicron variant. Our estimate of the omicron-induced prevalence of olfactory dysfunction in populations of European ancestry is 11.7%, while it is significantly lower in all other populations, ranging between 1.9% and 4.9%. When ethnic differences and population sizes are considered, the global prevalence of omicron-induced olfactory dysfunction in adults is estimated to be 3.7%. Omicron’s effect on olfaction is twofold to tenfold lower than that of the alpha or delta variants according to previous meta-analyses and our analysis of studies that directly compared the prevalence of olfactory dysfunction between omicron and previous variants. The profile of the prevalence differences between ethnicities mirrors the results of a recent genome-wide association study that connected a gene locus encoding an odorant-metabolizing enzyme, UDP glycosyltransferase, to the extent of COVID-19-related loss of smell. Our analysis is consistent with the hypothesis that this enzyme contributes to the observed population differences.

## 1. Introduction

The omicron variant has been reported to cause less anosmia than the preceding SARS-CoV-2 virus variants [1,2,3,4]. Since the prevalence of olfactory dysfunction varies greatly between studies, the global prevalence of anosmia caused by omicron has not yet been estimated. The number of confirmed COVID-19 cases reported to the World Health Organization (WHO) by 30 November 2022, was 639 million (WHO Coronavirus (COVID-19) Dashboard, https://covid19.who.int/, accessed on 1 December 2022), but the true number of cases is believed to be much higher, at about 3.4 billion in October 2021 [5]. A total of 6 billion cases—after the global spread of the more infectious omicron variant—was estimated in October 2022 [6]. Since the prevalence of olfactory dysfunction differs between virus variants [1,2,3,4,7,8], it is important for estimates of the current global and regional prevalence of olfactory dysfunction to account for the properties of different virus variants. It has been argued that even though omicron may cause lower prevalence of olfactory dysfunction, its increased infectivity may produce an equivalent number of—or even a net gain in—the cases of hyposmia or anosmia, because many more people will become infected with the omicron variant [9,10].

It is possible that host factors also contribute to the populational differences in COVID-19-related olfactory dysfunction [7,11,12,13]. Such host factors, besides age and gender, are apparently not related to differences in expression levels or in the genetic variation of the virus entry proteins, namely, ACE2 and TMPRSS2, as was initially assumed [11,12,14]; rather, they may be related to genetic variation and the frequency of risk alleles of an odorant-metabolizing enzyme, specifically, a glycosyltransferase that is encoded by the *UGT2A1/A2* locus [13]. This enzyme is abundantly expressed in the sustentacular support cells of the olfactory epithelium of vertebrates [15,16], including humans [17,18,19,20].

In this study, we conducted a systematic review and meta-analysis of the literature on olfactory dysfunction caused by the omicron variant. In this review, we focused on loss of smell rather than loss of taste. Loss of taste is thought to be, in part, due to loss of smell [21]; therefore, we grouped the diverse reports on “loss of smell”, “loss of smell and taste”, and “loss of smell or taste” into one category. Furthermore, because the vast majority of reports use patients’ subjective recall to identify new cases of olfactory dysfunction, we restricted our analysis to studies that used subjective methodology (the patient’s recollection of changes in smell) as opposed to objective psychophysical testing, which depends on cultural context and, therefore, requires population-specific validation [22]. Regarding omicron-associated olfactory dysfunction, we found 62 studies reporting on the basis of the patient’s subjective recall, and only one study that also performed psychophysical testing.

We generated estimates of the global prevalence of omicron-induced olfactory dysfunction, as well as regional prevalence, which is determined at least in part by genetics (prevailing ethnicity) within populations. The similarities between the results of our analysis and those of a recent genome-wide association study [13] indicate differences in the frequency of the risk allele for an odorant-metabolizing enzyme as a contributing factor, resulting in population differences in the prevalence of olfactory dysfunction.

## 2. Methods

### 2.1. Search Strategy

**Figure 1:**
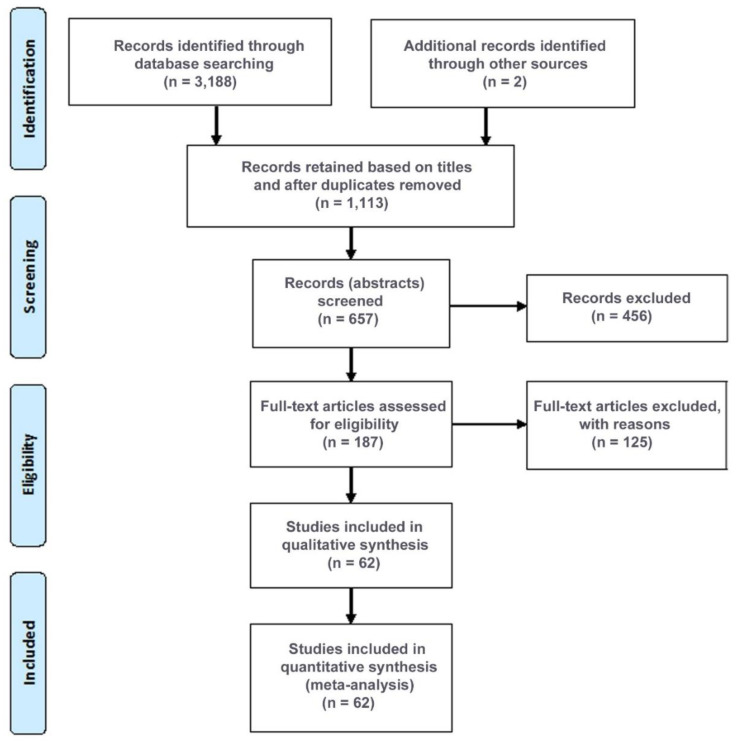
Flowchart illustrating the literature search, systematic review, and meta-analysis according to the PRISMA guidelines. PubMed, the NIH COVID-19 portal, and Google Scholar were searched systematically; other sources were found in references of articles and in news media. The literature was last updated on 10 January 2023. Given in decreasing frequency, the reasons for exclusion of full-text articles were as follows: no prevalence data; no data on omicron; long (not acute) COVID; children-only; review; and case report.

For our systematic review of the literature, we adhered to the guidelines set forth by the Preferred Reporting Items for Systematic Reviews and Meta-Analyses (PRISMA) [23]. Reports of studies that estimate the prevalence of olfactory dysfunction were identified through a search of two databases, namely, PubMed and Google Scholar, as well as the iCite NIH COVID-19 portal (https://icite.od.nih.gov/covid19/search/, accessed on 10 January 2023), for the years 2021, 2022, and 2023. The COVID-19 portal was included in order to capture preprints in addition to peer-reviewed articles. The following search strategy was formulated using the keywords “omicron” and “smell”, as well as “omicron” and “anosmia.” Only English terms were used in the search strategy. Reference lists from the eligible articles were examined to identify additional relevant studies. Duplicates were removed; however, in the case of preprints, the first date of publication was recorded, even when the peer-reviewed version of the paper, when available, was compiled in our list of references. All titles were screened, and when potentially relevant, the abstracts were evaluated to decide whether such a paper should be short-listed for full-text reading. The full texts of all short-listed records were reviewed to determine whether they were eligible according to our inclusion and exclusion criteria; then, they were used to produce the final selection of studies for inclusion in subsequent analyses (Figure 1). 

### 2.2. Inclusion/Exclusion Criteria

Studies that were deemed eligible for the systematic review met all of the following inclusion criteria: (1) studies reporting the numerical prevalence of olfactory dysfunction in humans infected with the omicron variant (B.1.1.529) and any of the omicron subvariants, namely, BA.1, BA.2, BA.1.1, BA.2.2, BA.2.10, BA.2.38, BA.2.75, BA.5, BQ.1, and XBB— studies solely reporting odds ratios only were not sufficient for inclusion [24]; (2) studies on adults or adolescents (when a small number of children was included, this was considered acceptable), but studies that focused entirely on children were not included, because it is known that children with COVID-19 have significantly lower prevalence of olfactory dysfunction than adults with COVID-19 [25]; (3) evidence of infection with SARS-CoV-2; genomic proof of variant type was not deemed necessary when it was known that the vast majority of infections during the period and in the region of data collection were omicron cases rather than cases caused by another virus variant; (4) olfactory dysfunction was monitored through subjective recall, and all members of the cohort were specifically asked about changes in smell, changes in smell or taste, or changes in smell and taste—review of medical records for entries regarding loss of smell, but without universal and specific questioning of patients, was not acceptable (e.g., [26]); and (5) the olfactory dysfunction occurred during the acute phase of infection; long-term studies inquiring about changes of smell persisting for weeks or months after the infection were not included. Comparison with variants other than omicron was not a required inclusion criterion.

### 2.3. Quality Assessment and Publication Bias

Risk of bias in cohort studies was assessed using a modified Newcastle–Ottawa scale (adapted Cochrane’s risk of bias tool [27]). This scale attempts to assess accuracy of measurements and whether the cohort is representative of the community. Duration of follow-up is not relevant for the current review and analysis. The modified scale assesses study design and cohort size, as well as information about convenience samples and response rates, when applicable. In addition, we explored the magnitude of the potential bias caused by survey-type studies that rely on the initiative and motivation of respondents [9,28,29,30] by comparing the results of traditionally design studies with those of survey-type studies. In addition, we generated funnel plots to assess potential publication bias [31].

### 2.4. Data Extraction

The relevant data of each study were extracted using pre-designed tables; such data included the first date of publication, first author, country, geographic region, the cohort size, the number of cases, and the percentage calculated from the number of cases per cohort. When applicable, the comparator virus variant was also noted, along with its cohort size, number of cases with olfactory dysfunction, and the percentage of prevalence, as well as the name(s) of the previous variant or variants causing the infection. When the comparator virus variant was not disclosed, it was retrieved as G614 vs. D614 [32]. Additional information about cohorts such as age, gender, and ethnic composition was recorded when studies provided this information. 

### 2.5. Subgroup Analyses and Comparisons

The global prevalence of olfactory dysfunction due to omicron infection was calculated by taking ethnic differences and population sizes into account, and this prevalence was compared with the global and regional prevalence due to previous variants using information from studies that reported such data (32 out of 62 studies). Due to ethnic differences between populations, the prevalence for each major ethnicity (European ancestry, African, Middle East, East Asian, South Asian, and Latino/Hispanic) was estimated separately and weighted by population size to calculate an estimate of the current global prevalence of hyposmia due to omicron. This was necessary to prevent bias since the largest fraction of available studies and those with the largest cohorts focused on people with European ancestry.

### 2.6. Data Synthesis

The primary purpose of the meta-analysis was to produce a more precise and reliable estimate of the effect of the omicron variant on olfactory dysfunction, compare this estimate with previous estimates that were made before the emergence of omicron (results from previous meta-analyses), and prepare a direct comparison by compiling the data from those studies that provided internal comparative data on other virus variants. 

### 2.7. Statistical Analyses

Pooled analyses were performed to determine olfactory dysfunction prevalence and risk ratio (RR). The heterogeneity among studies was evaluated by Cochran’s Q test and the I^2^ index [33,34]. Random-effect models were used to conservatively diminish the heterogeneity between the studies [34]. A continuity correction of 0.5 was applied to studies with zero cases [35]. The study weights were obtained based on the DerSimonian–Laird method [36]. Pooled analyses for subgroups were conducted according to ethnicity and study type (survey-type studies and traditional-design studies). Meta-regression analyses were performed to test the association between prevalence and key variables [35], including the UGT2A1 risk allele frequency [13] and the study type [36]. The risk of publication bias was evaluated using funnel plots and Egger’s test [37]. The significance level was set to 0.05. All the meta-analyses were performed using Stata SE 16.0 software (StataCorp, College Station, TX, USA).

## 3. Results

### 3.1. Properties of Studies

**Figure 2:**
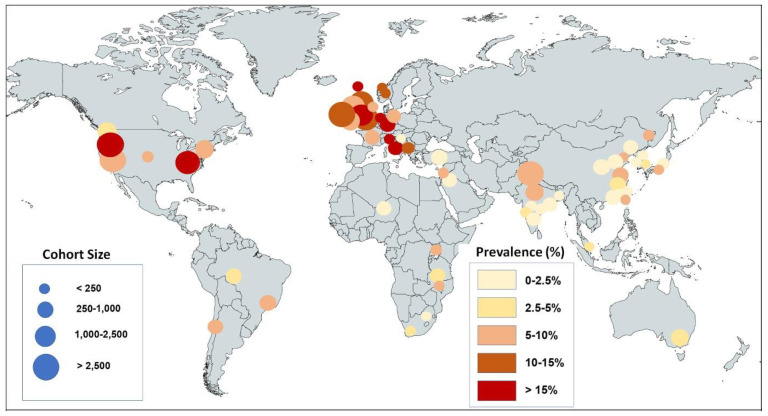
World map showing the locations of cohorts included in the systematic review and the prevalence of olfactory dysfunction caused by the omicron variant. The sizes of the circles represent the size of the cohort as indicated in blue; the color gradient indicates the prevalence range as shown on the right side. Note that populations of European ancestry have larger prevalence levels than populations of non-European ancestry.

We found 62 studies—published between 27 November 2021, and 10 January 2023—that met our inclusion criteria. Collectively, these studies reported the olfactory status of 626,035 patients infected with the omicron variant (Table 1). These studies were conducted in 26 countries on 6 continents (Figure 2). Twenty-five studies were on populations of primarily European ancestry [9,30,38,39,40,41,42,43,44,45,46,47,48,49,50,51,52,53,54,55,56,57,58,59,60], seventeen studies were on East Asians [61,62,63,64,65,66,67,68,69,70,71,72,73,74,75,76,77,78], (note that one of these studies, [75], reports on the same cohort as [74], and, therefore, was removed from the meta-analysis), eight studies were on South Asians [79,80,81,82,83,84,85,86], four studies were on Latinos/Hispanics [87,88,89,90], five studies were on populations in Africa [91,92,93,94,95], and three studies were on populations from the Middle East [96,97,98]. The locations of the studies, with the prevalence indicated by the color intensity, and the cohort size, indicated by the size of the circles, shows that Western countries reported the highest prevalence, while studies from East Asia and the Middle East reported the lowest prevalence (Figure 2). We found that 5 studies were of low quality, 36 of moderate quality, and 21 of high quality according to the modified Newcastle–Ottawa scale [27]. Thirty-two of the sixty-two studies also reported the olfactory status affected by one or more of the previous SARS-CoV-2 virus variants, mostly the delta variant (Table 2). 

### 3.2. Global Prevalence of Olfactory Dysfunction

**Figure 3:**
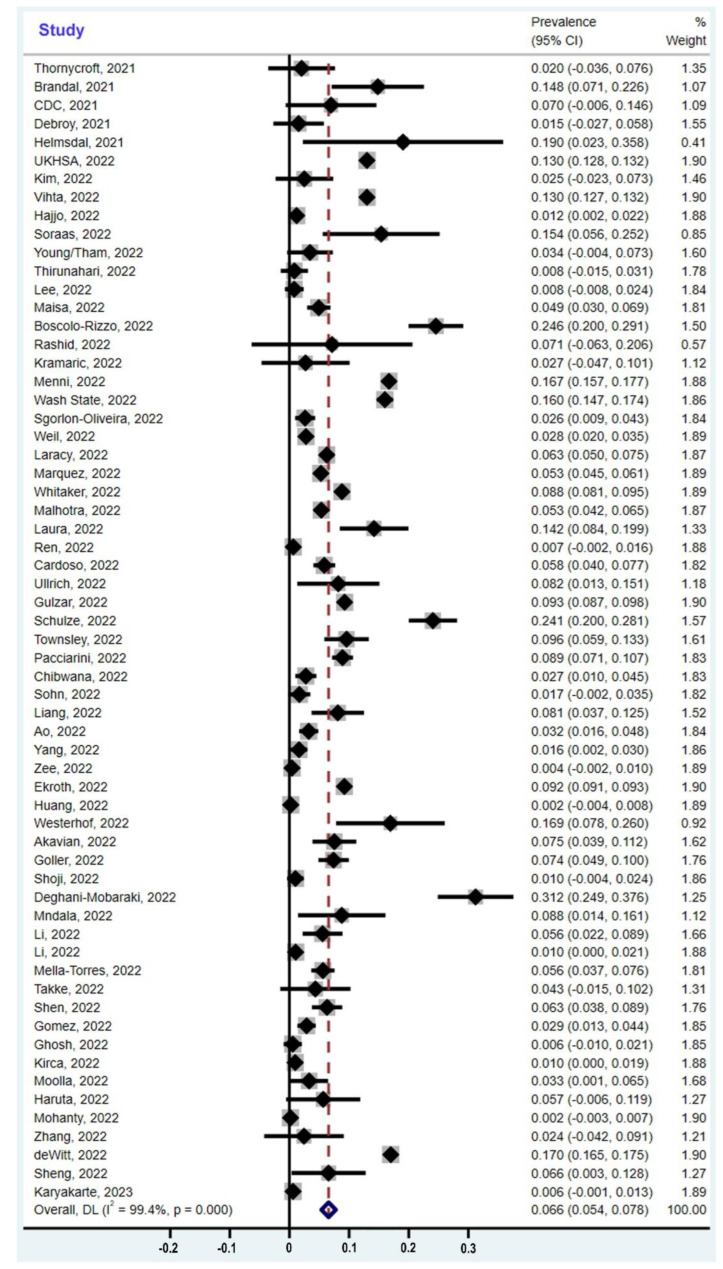
Forest plot of the 62 studies reporting the prevalence of olfactory dysfunction due to the omicron variant. The confidence intervals (CI) and the weight of each study are indicated on the right. The pooled overall global prevalence is 6.6% according to the meta-analysis, but this does not consider ethnic differences and population sizes as explained in Figure 4. The size of the light-grey box is proportional to the study weight. The study weights are obtained based on the DerSimonian–Laird method. CI, confidence interval; DL, DerSimonian–Laird method; I^2^, I-squared index.

**Figure 4:**
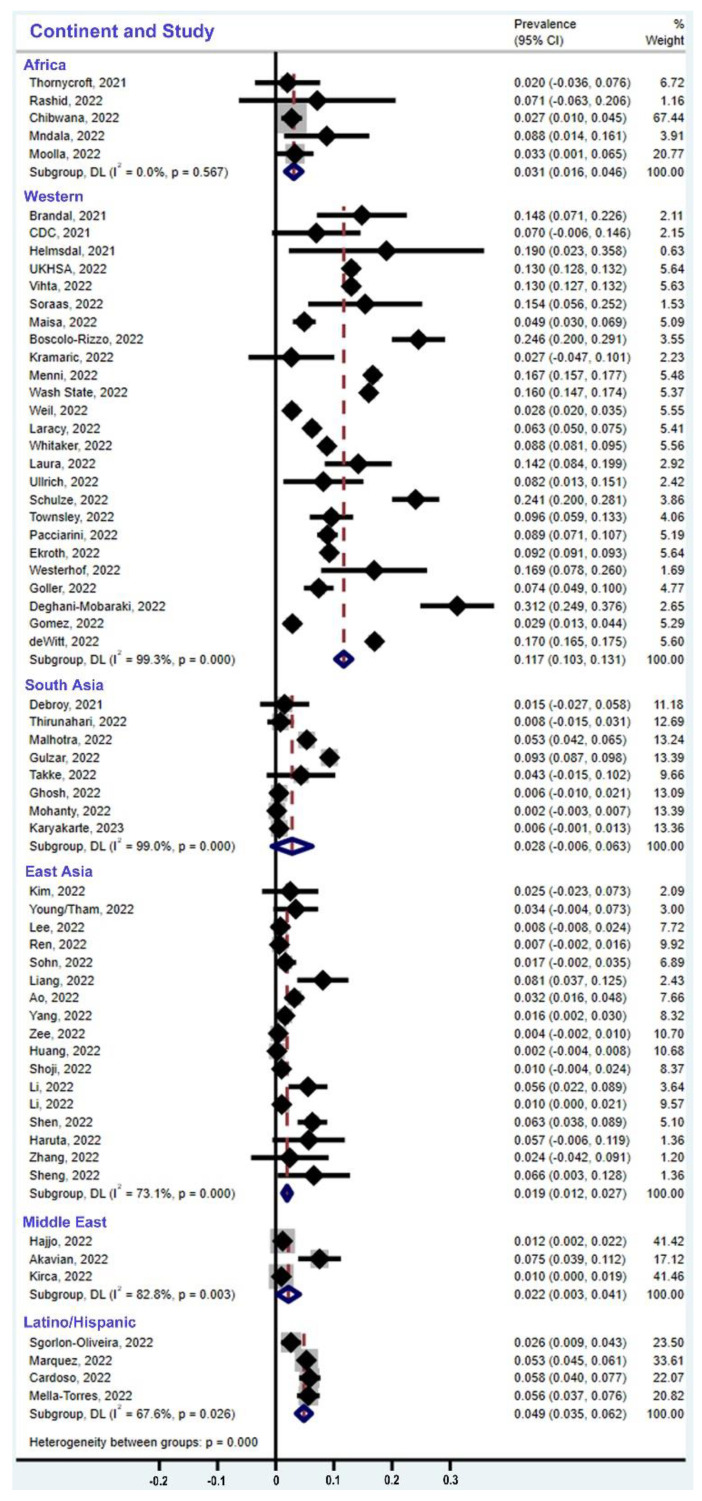
Forest plots of the prevalence of olfactory dysfunction due to omicron by different regions/ethnic populations according to the meta-analysis. Prevalence levels are as follows: 3.1% among populations in Africa (CI = 1.6–4.6%), 11.7% among people of European ancestry (Western countries, CI = 10.3–13.1%), 2.8% in South Asia (CI = 0–6.3%), 1.9% in East Asia (CI = 1.2–2.7%), 2.2% in the Middle East (CI = 0.3–4.1%), and 4.9% among Latinos/Hispanics (CI = 3.5–6.2%). The size of the light-grey box is proportional to the study weight. The study weights are obtained based on the DerSimonian–Laird method. CI, confidence interval; DL, DerSimonian–Laird method; I^2^, I-squared index.

When we combined all the eligible studies in the Forest Plot (Figure 3), we derived the following estimate of the global prevalence of olfactory dysfunction due to the omicron variant: 6.6% of adults are infected with this variant. However, this estimate obscures the effect of ethnicity as a major factor. Our meta-analysis of the studies reporting on populations of European ancestry, which are the majority of studies and the ones with the largest cohort sizes, shows that the pooled prevalence of olfactory dysfunction is 11.7% (Figure 4). On the other hand, populations of non-European ancestry have a much lower prevalence, ranging from 1.9% to 4.9%, as detailed below (Figure 4). When ethnic differences between populations and the current population sizes are weighted appropriately (Figure 4; Table 3), the global prevalence of olfactory dysfunction due to the omicron variant reduces to 3.7% of omicron-infected adults.

**Figure 5:**
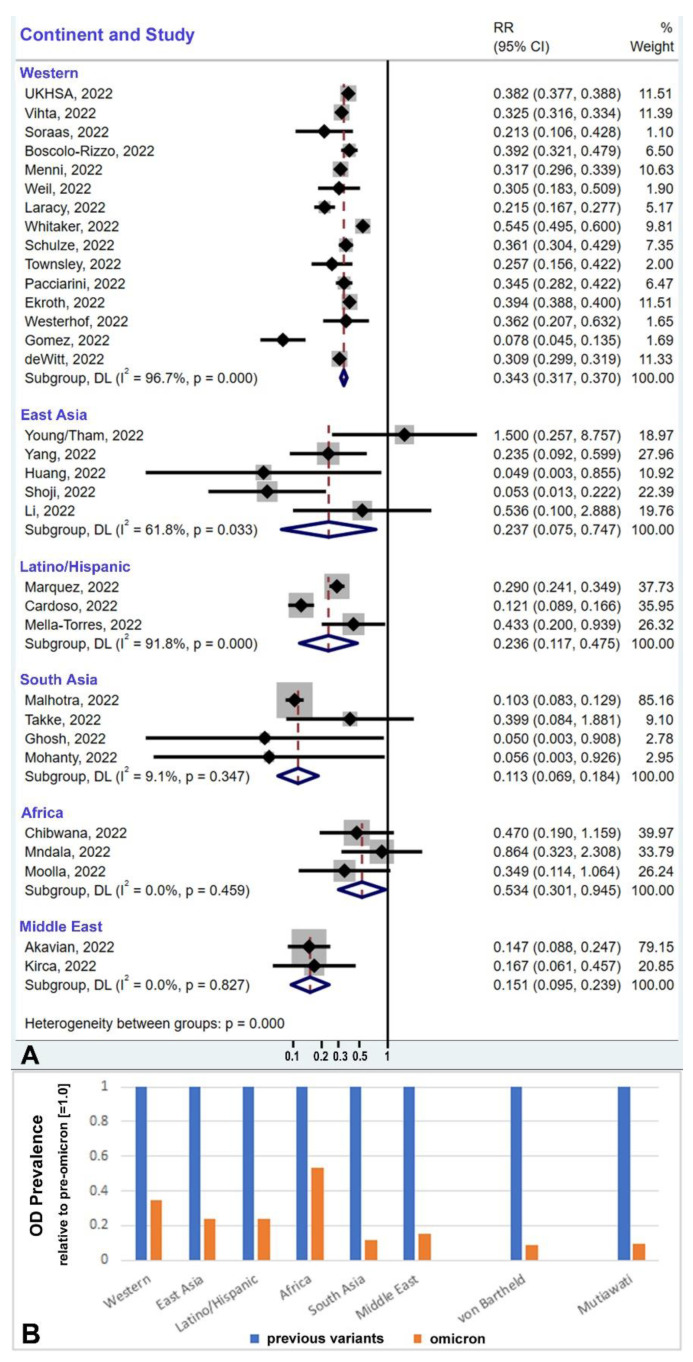
The prevalence of olfactory dysfunction (OD) due to omicron is reduced by 2-fold to 10-fold compared to the previous SARS-CoV-2 variants, regardless of ethnic population and region. (**A**) The reduction in OD due to omicron in direct comparison within similar populations and regions during the outbreak of the delta variant, predominantly (for specifics of the comparator variants, see Table 2). The size of the light-grey box is proportional to the study weight. (**B**) The bar graph summarizes the reduction in prevalence of OD for the direct comparisons from panel A (n = 32), and two indirect comparisons with pooled estimates from previous meta-analyses, namely, von Bartheld et al., 2020 [99] and Mutiawati et al., 2021 [100]. The percentage of reduction ranges between 2-fold and 10-fold. CI, confidence interval; DL, DerSimonian–Laird method; I^2^, I-squared index, RR, risk ratio.

**Figure 6:**
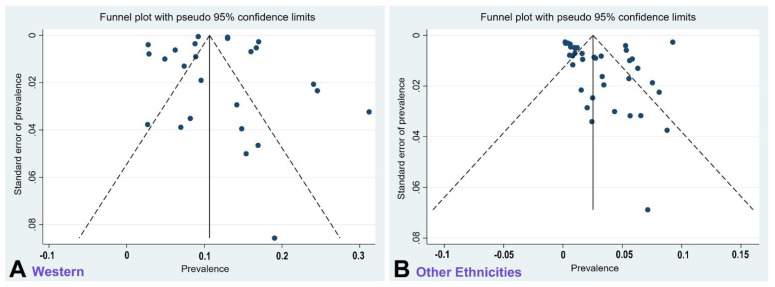
The funnel plots for the Western studies (**A**) and the studies for all other ethnicities (**B**) reporting omicron-induced olfactory dysfunction. These are scatterplots of prevalence levels against their standard errors. The vertical solid line is the estimated effect size; the dotted lines are the corresponding pseudo 95% confidence intervals (CIs). They provide insight into the spread of observed effect sizes. The majority of studies are randomly scattered within the CI region, indicating absence of publication bias for Western studies (**A**, *p* = 0.591) and for all other ethnicities (**B**, *p* = 0.703).

When we compared the prevalence of olfactory dysfunction due to omicron with that of previous variants (mostly delta), we found twofold to tenfold lower prevalence with omicron based on the thirty-two studies that provided a direct comparison (Table 2; Figure 5A,B). The overall reduction in olfactory dysfunction associated with omicron vs. previous variants is 0.299 (confidence intervals (CIs): 0.276, 0.323). This difference is statistically significant (*p* < 0.001). When compared with previous meta-analyses reporting on multiple SARS-CoV-2 variants up to August 15, 2020 (prevalence: 43.0%; 104 studies with 38,198 patients [99]) and up to November 10, 2020 (prevalence: 38.2%; 107 studies with 32,142 patients [100]), the prevalence of olfactory dysfunction due to omicron is 10-fold lower. The funnel plots (Figure 6A,B) indicate that the included studies do not demonstrate publication bias (A, *p* = 0.591; B, *p* = 0.703).

### 3.3. Geographic/Ethnic Differences

The studies compiled in Figure 2 and Figure 4 suggest that geography or ethnicity are relevant variables. Most ethnicities are well represented, with a robust number of studies and cohort sizes for Western countries (mostly people of European ancestry, n = 25 studies, with 602,089 people in all cohorts), for South Asia (n = 8 studies, with 14,165 people in all cohorts), East Asia (n = 17 studies, with 3585 people in all cohorts), and for the cohort of Latinos/Hispanics (n = 4 studies, with 4542 people in all cohorts). The population data are sparse for the African continent (n = 5 studies, with 544 people in all cohorts) and in the Middle East (n = 3 studies with 1100 people in all cohorts). A comparison of the subgroups indicates that omicron causes hyposmia prevalence levels of 1.9% in East Asia (CI = 1.2–2.7%), 3.1% in Africa (CI = 1.6–4.6%), 2.2% in the Middle East (CI = 0.3–4.1%), 2.8% in South Asia (CI = 0–6.3%), 4.9% in Latinos/Hispanics (CI = 3.5–6.2%), and 11.7% in people of European ancestry (Western countries, CI = 10.3–13.1%) (Figure 4). Since these data are derived from people infected with the same virus variant, the population difference must be primarily due to host factors rather than virus factors, as detailed in the Discussion section (Section 4).

### 3.4. Global Prevalence Considering Ethnic Differences and Population Sizes

When we account for the omicron-caused prevalence of hyposmia among the different major ethnicities, apply the total population sizes of these major ethnicities (obtained from the WHO website: https://www.worldometers.info/geography/7-continents/, accessed 10 January 2023), and use the estimated numbers of COVID-19 cases from the Institute for Health Metrics and Evaluation [6], we can estimate the number of adults in different ethnic populations that can be expected to experience olfactory dysfunction due to omicron infection (Table 3). Since children make up approximately 25% of the world population, we subtracted 25% from each of the population sizes to account for children, who are not included in our review because there are too few studies reporting on olfactory dysfunction in omicron-infected children, and children with COVID-19 are known to have far more reduced levels of olfactory dysfunction than adults [25]. Assuming a COVID-19 infection proportion of 90% among populations [6], we predict a total number of 71.1 million adults with hyposmia out of 0.6 billion for people of European ancestry (11.7% prevalence), 23.2 million adults with hyposmia out of 0.5 billion Latinos/Hispanics (4.9% prevalence), 23.9 million adults with hyposmia out of 1.35 billion South Asians (3.8% prevalence), 31.3 million adults with hyposmia out of 0.9 billion Africans (3.1% prevalence), 32.1 million adults with hyposmia out of 1.7 billion East Asians (1.9% prevalence), and 7.4 million adults with hyposmia out of 0.3 billion in the Middle East (2.2% prevalence), totaling 200.9 million adult people with olfactory dysfunction, as summarized in Table 3. The estimates for East Asians consider that the “Zero-COVID” policy in China has ended, meaning that China is expected to have a 90% infection rate, as estimated for the rest of the world [6]. The estimated numbers for East Asia would have been substantially lower during the implementation of the “Zero-COVID” policy in China.

### 3.5. Ethnic Profiles: Omicron-Induced Hyposmia vs. UGT2A1 Risk Allele Frequency

Initially, it was thought that differences in expression levels or in the genetic variation of the virus entry proteins ACE2 and TMPRSS2 may be host factors that contribute to population differences in COVID-19-induced olfactory dysfunction [11,12]. However, this hypothesis appears to be inconsistent with recent data [14], and it is now thought that the host factor is most likely an odorant-metabolizing enzyme, namely, a glycosyltransferase that is encoded by the *UGT2A1/A2* locus, based on a recent genome-wide association study showing significant ethnic differences in the frequency of the risk allele at this locus [13].

**Figure 7:**
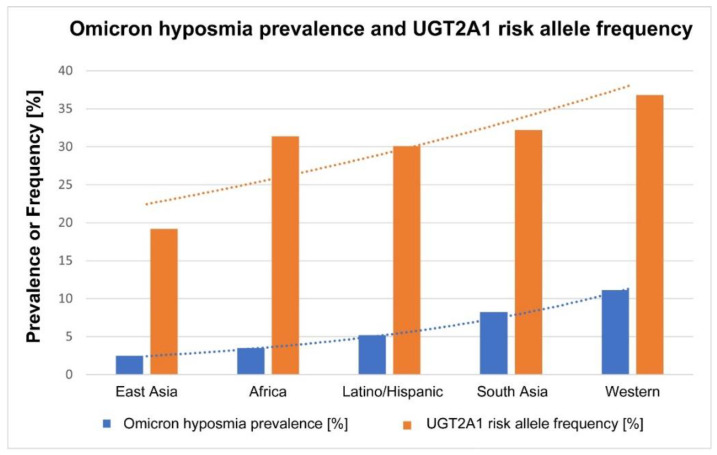
The bar graph shows the differences between ethnicities regarding omicron-induced prevalence of olfactory dysfunction (blue bars) compared with the frequency of the risk allele for olfactory dysfunction in the UGT2A1 locus according to Shelton et al., 2022 [13] (orange bars). The blue bars show the pooled estimates for the hyposmia prevalence among the same ethnicities according to our systematic review. Meta regression shows that there is a positive association between the two parameters (*p* < 0.001): the prevalence of olfactory dysfunction is higher in those ethnic populations that have a higher frequency of the risk allele. The two exponential trend lines show this similarity.

The ethnicity profile (East Asian, African, South Asian, Latino/Hispanic, and people with European ancestry) for both the risk allele frequency as well as hyposmia prevalence is shown in Figure 7. Our comparison of the major ethnicities for omicron-induced hyposmia prevalence reveals a remarkably similar ethnic profile when compared with the pattern described [13] for the frequency of the risk allele in the *UGT2A1* locus (Figure 7). We used meta regression to test whether the risk allele in the *UGT2A1* locus predicted omicron-induced hyposmia prevalence and we found that there is an association between a population’s risk allele frequency and omicron-induced hyposmia prevalence (*p* < 0.001). The coefficient is positive, which means that hyposmia prevalence is higher when the risk allele frequency is higher, which is consistent with a genome-wide association analysis [13]. This supports the idea that the odorant-metabolizing enzyme, the UDP glycosyltransferase, is involved as a host factor in the susceptibility to SARS-CoV-2-induced olfactory dysfunction.

### 3.6. Comparison of Survey-Type Studies and Traditional-Design Studies

**Figure 8:**
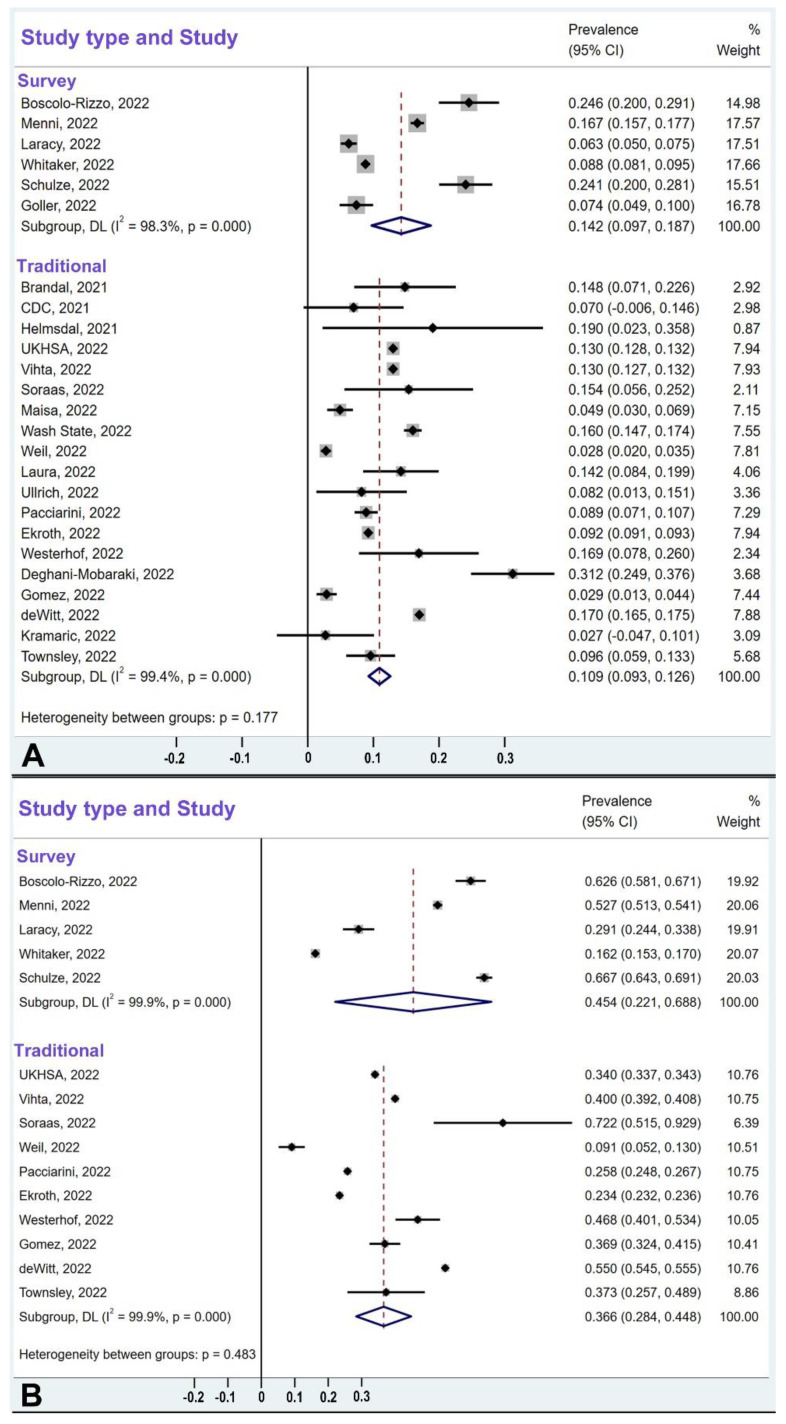
The pooled prevalence levels of olfactory dysfunction due to omicron (**A**) and due to other variants (**B**) in survey-type studies and traditional-design studies for populations of European ancestry. While the pooled estimates are higher in the survey-type studies than in the traditional-design studies, namely, 14.2% vs. 10.9% for omicron studies (**A**) and 45.4% vs. 36.6% for previous variants (**B**), meta-regression showed no heterogeneity between the two study types. Confidence intervals for omicron studies (**A**) were 9.7–18.7% for survey-type studies and 9.3–12.6% for traditional design studies. Confidence intervals for studies on previous virus variants (**B**) were 22.1–68.8% for survey-type studies and 28.4–44.8% for traditional design studies.

It has been cautioned that survey-type studies (which invite people to respond to questionnaires, often ones posted on the internet) may incorporate bias because people with more severe conditions tend to be more motivated to respond [9,28,29,30,36]. Therefore, we estimated the magnitude of such a potential bias by comparing survey-type studies with traditional-design studies with respect to people with European ancestry, considering both omicron-caused hyposmia as well as hyposmia caused by previous SARS-CoV-2 variants separately (Figure 8A,B). Consequently, we found that with omicron, the survey-based studies resulted in a pooled estimate of hyposmia prevalence corresponding to 14.2% (CI: 9.7–18.7%), which is higher than the 10.9% (CI: 9.3–12.6%) resulting from the traditional studies (Figure 8A). However, a meta regression analysis showed that there is no statistically significant difference between these two prevalence levels (*p* = 0.391). With the previous virus variants, mostly delta, the survey-based studies resulted in a pooled estimate of hyposmia prevalence equaling 45.4% (CI: 22.1–68.8%), which is higher than the 36.6% (CI: 28.4–44.8%) resulting from the traditional studies (Figure 8B). Again, a meta regression analysis showed that there is no statistically significant difference between these two prevalence levels (*p* = 0.428).

## 4. Discussion

### 4.1. Global Prevalence of Olfactory Dysfunction Caused by Omicron Variant Infection

We estimate the global prevalence of omicron-induced olfactory dysfunction in adults to be 3.7%. This estimate accounts for ethnic differences and population sizes and is based on the notion that 90% of the population has been or will be exposed to SARS-CoV-2 [6]. Our estimate of 3.7% prevalence translates into 200.9 million adults who can be predicted to experience omicron-induced olfactory dysfunction (Table 3). Our review and meta-analysis show that the prevalence of olfactory dysfunction after omicron infection is about 2–10-fold lower than with previous variants, with a substantial reduction in all ethnicities (Figure 5B).

Our analysis reveals significant ethnic differences in the prevalence of omicron-induced olfactory dysfunction. The estimation of the current prevalence level of omicron-induced olfactory dysfunction in Western countries of 11.7% is well supported, and the estimates for South Asians, Latinos/Hispanics, and East Asians, with 14,165, 4542, and 3585 people in the cohorts, respectively, are also fairly well attested. Our estimates for the prevalence in Africa (3.1%) and the Middle East (2.2%) are less certain, as they are based on only 5 and 3 studies, respectively, with 544 and 1100 people in the cohorts.

### 4.2. Why Is Omicron’s Effect on Olfaction Different than That of Previous Variants?

Two main reasons have been proposed to explain this phenomenon, and they are not mutually exclusive. The mutations in the spike protein make the omicron variant more hydrophobic [101], which may reduce the solubility of the virus in the mucus, thereby diminishing its ability to reach the olfactory epithelium [2,102]. Second, due to reduced furin cleavage, the omicron variant prefers an endosomal route via cathepsin for entry into host cells rather than a surface membrane fusion via the protease TMPRSS2 [103]. Sustentacular cells and Bowman gland cells are the cells in the olfactory epithelium that most abundantly express both ACE2 and TMPRSS2 [104,105]; therefore, these support cells were the prime target of previous SARS-CoV-2 variants for host cell entry via cell surface membrane fusion enabled by TMPRSS2 [106]. Since the support cells—similar to many other host cells—have evolved more potent defense mechanisms for the endosomal route of infection [102,107], for example, the antiviral *IFITM2* gene is the most highly upregulated gene in support cells at 3 days after infection [108], this may lead to a lower efficiency in omicron infection of the support cells in the olfactory epithelium, and, therefore, reduced olfactory dysfunction [102,109].

### 4.3. Ethnic Differences in UGT2A1 Risk Allele Frequency: Implications

The similarity in the ethnic profiles between omicron-induced prevalence of hyposmia and the frequency of the *UGT2A1* risk allele (Figure 7) suggests that the *UGT2A1*-encoded glycosyltransferase is the host factor, or one of the major host factors, that determines the risk of olfactory dysfunction due to SARS-CoV-2 infection. How does the UDP glycosyltransferase affect the sense of smell? This is an evolutionarily highly conserved enzyme related to olfaction, not only in rodents and humans [16,18], but also in invertebrates [15]. It is thought to modulate the concentration of odorant molecules and terminate odorant signal transduction [17,110]. It contributes to the biotransformation of odorant molecules, prevents saturation of the odorant receptors, modifies the perceived quality of odorants [16,110], and thereby plays a major role in olfactory sensitivity. Polymorphisms in the enzyme may account for inter-individual variability in olfactory perception [16]. Furthermore, UDP glycosyltransferases are expressed differentially with aging [110], which could explain the increased olfactory dysfunction seen in young adults (and reduced dysfunction in children or older people [25,99]), and the expression of UDP glycosyltransferase also differs between genders [111], which may explain the higher susceptibility to olfactory dysfunction among females [99].

What does the genetic/ethnic difference in the risk allele frequency in the host (with the most extreme values in East Asians vs European ancestry) tell us about olfactory dysfunction? The risk allele at the *UGT2A1* locus causes a greater degree of olfactory dysfunction [13], which may explain why Europeans are more susceptible to a loss of smell, but the mechanism still is unclear. Nevertheless, the new data implicate the sustentacular cell as the site of pathogenesis, thus directing us towards a better understanding of how SARS-CoV-2 attacks the olfactory system.

### 4.4. Technical Considerations of Methodology

Similar to pre-omicron COVID-19 research wherein the large majority of studies reported olfactory dysfunction based on patient recall [27], we were forced to rely on studies reporting the results of such subjective testing or patient recall for the monitoring of olfactory dysfunction. In fact, we found only one study that reported omicron-induced hyposmia based on psychophysical testing [66], while there were 62 studies that reported on subjective recall (Table 1). It is currently controversial whether subjective recall or psychophysical testing is the most valid and sensitive approach to assessing COVID-related chemosensory dysfunction [21,27]. Among studies that compared the two types of methods, about half of them concluded that objective testing is more sensitive than subjective testing [112,113,114,115]. The other half concluded the opposite, i.e., that subjective testing is more sensitive than objective testing [116,117,118,119,120]. While some authors recommend that psychophysical testing is superior to subjective patient recall [121,122], others have pointed out that for the assessment of olfactory dysfunction during the COVID-19 pandemic, psychophysical testing by ENT specialists is largely impractical, because people with an acute COVID-19 infection typically quarantine during the acute phase. Furthermore, since chemosensory loss often lasts only about a week [99,123], smell and taste may recover before they can be tested quantitatively by experts [21]. Another argument against psychophysical testing is that its interpretation requires not only cross-cultural validation [22,124] but also a pre-pandemic or pre-infection base level for each individual because of the large fraction (nearly 30%) of people with pre-existing olfactory dysfunction in the normal population when using this method [125]. However, such a pre-pandemic base level is rarely available.

The vast majority of the studies we compiled were scored as moderate or high quality according to the modified Newcastle–Ottawa scale [27] (Table 1), and the omission of studies that were assessed to have low quality did not change our results and conclusions. There was no evidence for publication bias in the analysis of the funnel plots (Figure 6A,B). Since people who are more impacted by their condition may be more likely to respond to an internet-based survey [9,28,29,30,36], survey-type studies may contain bias. Therefore, we compared survey-type studies with traditional representative-sampling studies that use the direct and immediate questioning of each member of the eligible cohort rather than inviting eligible individuals online and collecting responses on internet-provided questionnaires. Such study designs rely on the equitable participation of individuals suffering from loss of smell and those without such an affliction. We found that although survey-type studies reported higher prevalence of hyposmia than those employing traditional study designs, there was no evidence for heterogeneity between the two study types (Figure 8A,B). The issue of potential bias in survey-type studies deserves further scrutiny and should be examined in the future with more studies and larger cohorts.

### 4.5. Limitations of our Review

Most studies compiled in our review were not stratified according to age group, but age is a relevant factor [13,99,126]. Likewise, most studies did not report on the gender of the cohort nor the gender of the cases, yet gender is also a relevant factor [13,99]. There were few studies from Africa and the Middle East, and those studies had small cohorts; thus, additional data are needed to conduct a more reliable subgroup analysis and achieve higher certainty with respect to the prevalence of hyposmia in these populations.

Many studies did not specify change in smell vs change in taste and reported them as either change or loss of smell *and* taste or change or loss of smell *or* taste. Additional studies are needed to better distinguish the effects of omicron on smell and taste.

We did not attempt to resolve whether omicron sub-variants have different effects on olfactory dysfunction; there are still too few studies that report the effects of subvariants on loss of smell [48,50,86].

Some cohorts of the studies were ethnically mixed, but the exact ethnic composition of the cohort was reported in only a few studies (e.g., [48]), and none of the studies reported the prevalence of olfactory dysfunction separately for distinct ethnicities. The latter should be performed in the future to verify differences between ethnicities, and this may also “sharpen” the ethnic distinctions that may be blurred in ethnically mixed cohorts. For example, the large fraction of Asians and/or Latinos/Hispanics in the cohorts of Weil et al. [48] and (likely) of Laracy et al. [49] may explain the relatively low overall prevalence of hyposmia in their studies. Although ethnic patterns are emerging, more detailed analyses in future studies may allow for the assignment of more precise values for the prevalence of olfactory dysfunction to each major ethnicity.

We did not include studies that focused on olfactory dysfunction in children. Children have lower hyposmia prevalence than adults [25], and the reduction in the prevalence with omicron would be difficult to quantify, given the considerable ethnic differences. Nevertheless, to our knowledge, there have been six studies that reported children’s prevalence of olfactory dysfunction due to the omicron variant [127,128,129,130,131,132], and all of these studies except for one [130] found a substantial reduction (about five-fold) in omicron-induced olfactory dysfunction when compared with previous variants, which is similar to the relative sparing of olfaction by omicron among adults (Figure 5).

### 4.6. Future Directions

Although we have a clue that the *UGT2A1* locus, and, therefore, the UDP glycosyltransferase, is involved in the ethnic differences in COVID-19-related olfactory dysfunction [13], the mechanism remains unclear. Nevertheless, the sustentacular support cells in the olfactory epithelium appear to play a major role. This finding helps direct focus on this cell type and its key roles with respect to the processing of odorants and the fundamental workings of the sense of smell [102]. A better understanding of the molecular mechanisms of loss of smell via COVID-19 infection may yield information about new therapies to help patients with persistent loss of smell beyond the current olfactory training, for which the latter is not effective in more than half of cases [133].

While some studies indicate that a previous SARS-CoV-2 infection may reduce the likelihood of olfactory dysfunction in a subsequent COVID-19 infection [51], it is known that a previous COVID-19 infection with an earlier variant does not necessarily prevent a second bout of loss of smell when the same individual is subsequently infected with a different SARS-CoV-2 variant [134,135]. It also does not seem that vaccinations reliably prevent the occurrence of loss of smell in breakthrough infections [9,52,88,136].

If the omicron variant infects about 90% of 6 billion adult people worldwide, what does a global prevalence of 3.7% olfactory dysfunction (200.9 million cases world-wide) mean for trends in global cases of olfactory dysfunction? Our ethnicity-adjusted projections suggest that olfactory dysfunction will decline globally despite the higher infectivity of the omicron variant, contrary to previous predictions [9,10]. It is not yet known whether there will be *persistent* loss of smell after omicron infection. Will it be similar to previous variants, with an about 5% level of persistent loss of smell among those who experience olfactory dysfunction [10]? Does a lower number of cases of olfactory dysfunction with omicron also reduce the percentage of those who will be afflicted by a persistent loss of smell? We have insufficient knowledge regarding persistent loss of smell caused by omicron, since it has been only little more than one year since the first cases of omicron infection emerged. Much is still to be learned about the effects of omicron (and previous and future) variants of SARS-CoV-2 on olfaction.

## Figures and Tables

**Table 1:** List of studies reporting the prevalence of olfactory dysfunction (OD) caused by the omicron variant.

Date First Published	Ref #	Author and First Publication Date	Cohort Country or Region	Cohort Size	Cases with OD	OD %	Quality Scores
27 November 2021	91	Thornycroft	South Africa	24	0	0.0%	L
16 December 2021	38	Brandal	Norway	81	12	14.8%	M
17 December 2021	39	CDC	USA	43	3	7%	M
17 December 2021	79	Debroy	India	32	0	0.0%	L
31 December 2021	40	Helmsdal	Denmark	21	4	19%	L
14 January 2022	41	UKHSA	UK	182,133	23,677	13%	M
17 January 2022	61	Kim	Korea	40	1	2.5%	M
18 January 2022	42	Vihta	UK	69,372	9018	13%	M
25 January 2022	96	Hajjo	Jordan	500	6	1.2%	M
27 January 2022	43	Soraas	Norway	52	8	15%	M
27 January 2022	62	Young/Tham	Singapore	87	3	3.44%	M
January 2022	80	Thirunahari	Telangana, India	60	0	0%	H
10 February 2022	63	Lee	Korea	123	1	0.8%	M
12 February 2022	44	Maisa	France	468	23	4.9%	H
18 February 2022	9	Boscolo-Rizzo	Italy	338	83	24.6%	H
28 February 2022	92	Rashid	Uganda, Africa	14	1	7.1%	H
2 April 2022	45	Kramaric	Slovenia	18	0	0.0%	M
6 April 2022	46	Menni	UK	4990	833	16.7%	H
13 April 2022	47	Washington State	WA, USA	2830	453	16%	L
28 April 2022	87	Sgorlon-Oliveira	Rondonia, Brazil	343	9	2.6%	M
28 April 2022	48	Weil	WA, USA	1730	48	2.8%	H
4 May 2022	49	Laracy	NY, USA	1520	95	6.3%	M
23 May 2022	88	Marquez	CA, USA	3032	160	5.3%	H
23 May 2022	50	Whitaker	UK	6395	563	8.8%	H
6 June 2022	81	Malhotra	New Delhi, India	1461	78	5.3%	H
13 June 2022	51	Laura	Bosnia	141	20	14.2%	H
16 June 2022	64	Ren	Tianjin, China	307	2	0.7%	L
24 June 2022	89	Cardoso	Brazil	633	37	5.8%	H
25 June 2022	52	Ullrich	Germany	61	5	8.2%	M
June 2022	82	Gulzar	Kashmir, India	11,715	1084	9.3%	M
1 July 2022	30	Schulze	Germany	428	103	24.1%	M
10 July 2022	53	Townsley	London, UK	240	23	9.6%	M
12 July 2022	54	Pacchiarini	Wales, UK	1000	89	8.9%	H
17 July 2022	93	Chibwana	Malawi, Africa	328	9	2.7%	H
19 July 2022	65	Sohn	Korea	181	3	1.7%	H
2 August 2022	66	Liang	Tianjin, China	148	12	8.1%	M
8 August 2022	67	Ao	Shanghai, China	465	15	3.2%	M
11 August 2022	68	Yang	Tianjin, China	310	5	1.6%	H
15 August 2022	69	Zee	Hong Kong, China	454	2	0.4%	M
17 August 2022	55	Ekroth	UK	309,912	28,569	13.4%	H
17 August 2022	70	Huang	Taiwan, China	224	0	0.0%	M
19 August 2022	56	Westerhof	Netherlands	65	11	16.9%	M
03 September 2022	97	Akavian	Israel	199	15	9.1%	M
9 September 2022	57	Goller	Germany	405	30	7.4%	M
11 September 2022	71	Shoji	Japan	199	2	1.0%	H
14 September 2022	58	Deghani-Mobaraki	Italy	205	64	31.2%	M
22 September 2022	94	Mndala	Malawi, Africa	57	5	8.8%	M
11 October 2022	72	Li	Jilin, China	180	10	5.6%	H
12 October 2022	73	Li	Henan, China	384	4	1.0%	M
21 October 2022	90	Mella-Torres	Chile	534	30	5.6%	M
31 October 2022	83	Takke	Mumbai, India	46	2	4.3%	M
7 November 2022	74	Shen	Shanghai, China	349	22	6.3%	H
16 November 2022	59	Gomez	Australia	452	13	3.2%	M
18 November 2022	84	Ghosh	Bangladesh	90	0	0.0%	M
24 November 2022	98	Kirca	Turkey	411	4	1.0%	M
24 November 2022	95	Moolla	South Africa	121	4	3.0%	M
2 December 2022	76	Haruta	Japan	53	3	5.7%	M
9 December 2022	85	Mohanty	Odisha, India	267	0	0.0%	H
9 December 2022	77	Zhang	Fujian, China	20	0	0.0%	M
15 December 2022	60	deWitt	NC, USA	19,189	3262	17%	M
16 December 2022	78	Sheng	Taiwan, China	61	4	6.6%	M
6 January 2023	86	Karyakarte	Pune, India	494	3	0.7%	H
				**Total:** **625,945**	**Total:** **68,545**		

**Footnotes:** Young/Tham: there were two different first authors on versions 1 and 2 of preprint server; quality scores according to the modified Newcastle–Ottawa scale [27]: L—low, M—moderate, and H—high. OD, olfactory dysfunction; Ref, reference; CA, California; NC, North Carolina; NY, New York; WA, Washington.

**Table 2:** Compilation of the 32 studies that compares prevalence of olfactory dysfunction due to omicron with that due to delta or other variants.

Region	Ref #	Author	Country or Region	Cohort Size	Percentage of Hyposmia	Cohort Size	Percentage of Hyposmia	Reduction Om./Prev	Variant Name
				Omicron		Previous Variants			
Middle East	97	Akavian	Israel	199	9.1%	119	51.3%	17.5%	G614
Middle East	98	Kirca	Turkey	411	1%	960	5.8%	17.2%	wt
Africa	95	Moolla	South Africa	121	3.3%	116	9.5%	34.7%	G614
Africa	93	Chibwana	Malawi	328	2.7%	154	5.8%	46.6%	δ?
Africa	94	Mndala	Malawi	57	8.8%	128	10.2%	86.3%	δ
East Asia	62	Young/ Tham	Singapore	87	3.44%	87	2.3%	149.6%	δ
East Asia	68	Yang	Tianjin, China	310	1.6%	422	6.9%	23.2%	β, δ
East Asia	73	Li	Jilin, China	384	1%	103	2%	50.0%	δ
East Asia	71	Shoji	Japan	199	1.0%	111	19%	5.3%	δ
East Asia	70	Huang	Taiwan, China	224	0.0%	141	4.3%	0.0%	α
South Asia	84	Ghosh	Bangladesh	90	0.0%	40	10.0%	0.0%	δ
South Asia	85	Mohanty	Odisha, India	267	0.0%	461	3.2%	0.0%	δ ?
South Asia	83	Takke	Mumbai, India	46	0.43%	55	10.9%	39.4%	δ
South Asia	81	Malhotra	New Delhi, India	1461	5.3%	1907	51.6%	10.3%	δ
Hispanic	88	Marquez	CA, USA	3032	5.3%	1533	18.2%	29.1%	δ, prev.
Latino	89	Cardoso	Brazil	633	5.8%	5420	48.2%	12.0%	wt, γ, δ
Latino	90	Mella-Torres	Chile	534	5.6%	54	13%	43.1%	δ
Western	41	UKHSA	UK	182,133	13%	87,920	34%	38.2%	δ
Western	42	Vihta	UK	69,372	13%	14,318	40%	32.5%	δ
Western	43	Soraas	Norway	52	15%	18	72.2%	20.8%	δ
Western	9	Boscolo-Rizzo	Italy	338	24.6%	441	62.6%	39.3%	G614
Western	46	Menni	UK	4990	16.7%	4990	52.7%	31.7%	δ
Western	48	Weil	WA, USA	1730	2.8%	209	11.1%	25.2%	δ
Western	49	Laracy	NY, USA	1520	6.3%	361	29%	21.7%	α, δ
Western	50	Whitaker	UK	6395	8.8%	6739	16.2%	54.3%	wt, α, δ
Western	30	Schulze	Germany	428	24.1%	1497	66.7%	36.1%	G614, α, δ
Western	54	Pacciarini	Wales, UK	1000	8.9%	8,168	25.8%	34.5%	δ
Western	55	Ekroth	UK	309,912	13.4%	123,529	33.7%	39.8%	δ
Western	56	Westerhof	Netherlands	65	16.9%	216	46.7%	36.2%	G614, α
Western	59	Gomez	Australia	452	3.2%	425	36.9%	8.7%	δ
Western	53	Townsley	London, UK	240	9.6%	67	37.3%	25.7%	δ
Western	60	deWitt	NC, USA	19,189	17%	37,711	55%	30.9%	pre δ

**Footnotes:** α, alpha variant; δ, delta variant; γ, gamma variant; G614, variant with the D to G mutation at position 614; Om., omicron; Prev., previous variants; Ref #, Reference Number; wt, wildtype. Young/Tham, two different first authors on versions 1 and 2 of preprint server; CA, California; NC, North Carolina; NY, New York; WA, Washington.

**Table 3:** Estimation of the number of adults of different ethnicities expected to experience olfactory dysfunction (OD) following infection with the omicron variant.

	Population	Adults Only	COVID-19 -Infected Adults *	OD Pre-Valence	Adults with OD	Weight	Prevalence × Weight
	Billion	Billion	Billion	%	Million		
Western	0.9	0.675	0.6075	11.7	71.1	0.11	1.32
Latino/Hispanic	0.7	0.525	0.4725	4.9	23.2	0.09	0.43
Africa	1.4	1.050	0.9450	3.1	29.3	0.18	0.54
East Asia	2.5	1.875	1.6875	1.9	32.1	0.31	0.59
South Asia	2.0	1.500	1.3500	2.8	37.8	0.25	0.70
Middle East	0.5	0.375	0.3375	2.2	7.4	0.06	0.14
Total	8.0	6.00	5.40		200.9	1.00	3.72

**Footnotes:** * COVID-infected = 90% for all populations (IHME, 21 October 2022 [6]); Weight = (number of COVID-19 patients in one continent)/(number of total COVID-19 patients).

## Data Availability

All data referenced in this study are publicly available.
